# Anti‐Apoptotic Effects of Escin on *Porphyromonas gingivalis*–Derived Lipopolysaccharide‐Induced Injury in SH‐SY5Y Cells

**DOI:** 10.1002/brb3.70810

**Published:** 2025-09-02

**Authors:** Yingying Sun, Rui Zhang, Yunping Pan, Yufei Sun, Limin Liu, Min Feng, Yuan Du, Tian Wang, Shutai Liu

**Affiliations:** ^1^ Department of Periodontology Shenzhen Stomatology Hospital Shenzhen Guangdong China; ^2^ Key Laboratory of Molecular Pharmacology and Drug Evaluation (Yantai University), Ministry of Education, School of Pharmacy Yantai University Yantai Shandong China

**Keywords:** apoptosis | escin | Parkinson's disease | *Porphyromonas gingivalis*

## Abstract

**Background:**

*Porphyromonas gingivalis*–derived lipopolysaccharide (*Pg*‐LPS) may be associated with the pathogenesis of Parkinson's disease and other neurodegenerative disorders. Escin is a bioactive compound found in the horse chestnut tree (*Aesculus hippocastanum*). This research investigated the impact of escin on *Pg*‐LPS‐induced injury in SH‐SY5Y cells.

**Methods:**

Parkinson's disease model was established using SH‐SY5Y neuronal cells. Cell viability was evaluated with MTT assays. Cell morphology was observed by fluorescence microscopy. Apoptosis was detected by JC‐1, TUNEL, and Hoechst/propidium iodide staining, and flow cytometric analysis. The mRNA levels of *BCL2* and *BAX* were determined by RT‐PCR, and the protein levels of *BCL2*/*BAX*, cleaved Caspase 3/Caspase 3, and PARP were evaluated through Western blotting.

**Results:**

Escin mitigated the reduction in viability of SH‐SY5Y cells caused by *Pg*‐LPS. *Pg*‐LPS triggered apoptosis in SH‐SY5Y cells, alleviated by escin dose‐dependently. Furthermore, the anti‐apoptotic effect of escin was reversed by a specific apoptotic inducer.

**Conclusions:**

*Pg*‐LPS leads to SH‐SY5Y cells’ mitochondria malfunction and programed cell death. Escin alleviated SH‐SY5Y cell injury induced by *Pg*‐LPS through its anti‐apoptotic effects.

## Graphical abstract

*Porphyromonas gingivalis*–derived lipopolysaccharide triggered apoptosis in SH‐SY5Y cells. However, escin attenuated this *P. gingivalis*–derived lipopolysaccharide‐induced cell injury. These findings provide an experimental basis for managing and preventing Parkinson's disease.

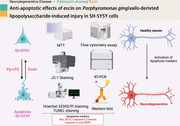

## 1 Introduction

Periodontitis is a prevalent, chronic infectious disease characterized by the destruction of the structures that support the teeth. This ultimately results in tooth decay. The prevalence of periodontitis is around 50% in adults, with 10% experiencing severe cases (Eke et al. 2015). *Porphyromonas gingivalis* is a significant causative agent of periodontitis and a contributing factor to systemic conditions, such as neurodegenerative disorders (Jungbauer et al. 2022). *P. gingivalis*–derived lipopolysaccharide (*Pg*‐LPS) is an important pathogenic factor that can trigger immune responses and inflammation (DeLegge and Smoke 2008).

For neurodegenerative disorders, the inflammation could be a non‐obvious but very important contributing factor (Skrzypczak‐Wiercioch and Sałat 2022). Lipopolysaccharide (LPS), an immunogenic particle in outer membrane of Gram‐negative bacteria, can trigger the inflammatory cascade in response to a Gram‐negative bacteria infection. Central LPS injection and systemic LPS challenge both have been used as models to elucidate Parkinson's disease (PD) (Batista et al. 2019). Emerging evidence indicates that periodontal pathogens may be an additional factor, beyond gut microbiota, that exacerbates PD. These pathogens and their metabolites have the potential to infiltrate the brain through pathways such as the trigeminal nerve and the blood–brain barrier (BBB), leading to neuronal damage (Ciccotosto et al. 2024). The inflammation induced by these pathogens can exacerbate the neurodegenerative progression seen in PD. Increased periodontal disease rates and the presence of related pathogens in brain tissues have been observed in PD patients. PD is 1.5 times more likely in subjects with chronic periodontitis compared with healthy subjects (Chen et al. 2017). Periodontal treatment significantly reduced systemic inflammatory markers in PD patients, indicating a potential therapeutic avenue (Martimbianco et al. 2021). Research in animal models indicates that eliminating *P. gingivalis* infections can mitigate neuroinflammation and enhance motor abilities in PD simulations (Ishida et al. 2017).

Causative agents of periodontitis, such as *P. gingivalis*, may be involved in PD development because of their effects on neuroinflammation, neuronal injury, and the clustering of harmful proteins such as α‐synuclein. Elevated levels of inflammatory markers have been observed in the trigeminal ganglia of individuals with chronic periodontitis, further supporting this hypothesis (Chen et al. 2017). *Pg*‐LPS can compromise the integrity of the BBB, either by triggering cell surface receptors or by directly inciting inflammatory processes within cells. This mechanism not only fuels neuroinflammation but also contributes to the deterioration of neurons (Verma et al. 2024). However, whether *P. gingivalis* can promote PD pathogenesis through other mechanisms, such as apoptosis, remains unclear.

Derived from the seeds of *Aesculus hippocastanum* (Chinese or Wilson's horse chestnut tree), escin is a potent triterpene saponin known for its therapeutic properties. This compound has demonstrated significant efficacy in mitigating central nervous system damage and cognitive decline through anti‐inflammatory, anti‐edema, and anti‐apoptotic effects (Selvakumar et al. 2020). Escin can mitigate ischemia–reperfusion brain damage in rodents by diminishing infarct size and water retention and by reducing neurological deficits (Wang et al. 2015). Additionally, escin can ameliorate neurological dysfunction by suppressing systemic inflammation (Ding et al. 2021). Escin showed a protective effect in PD through its anti‐apoptotic, antioxidant, and anti‐inflammatory properties (Selvakumar et al. 2020).

We hypothesized that *P. gingivalis* might contribute to the initiation and progression of PD via the activation of apoptotic pathways. With employed an in vitro SH‐SY5Y cell‐based model, whether escin could attenuate the *Pg*‐LPS‐induced injury in SH‐SY5Y cells was also investigated of PD to determine whether *Pg*‐LPS participates in neuronal cell injury. We then explored the therapeutic benefits of escin.

## 2 Materials and Methods

### 2.1 SH‐SY5Y Cell Culture

SH‐SY5Y cells (Cat. No. CL‐0208) were obtained from Wuhan Pricella Biotechnology Co. Ltd. (Wuhan, China). Cells were used at Passages 3–5 and confirmed mycoplasma‐free. The catalog number of the SH‐SY5Y cells used was CL‐0208. The cell provider performed mycoplasma testing and STR profiling. The mycoplasma test result was negative, and the STR profile did not reveal any contamination from other cell lines. *Pg*‐LPS was sourced from Sigma‐Aldrich (MO, USA). The cells were grown in a DMEM culture medium with high glucose (Hyclone, MA, USA). This medium was spiced up with 1% penicillin‐streptomycin (Gibco, MA, USA) and 10% fetal bovine serum (AusgeneX, Australia). Before experimentation, the cells were treated with 10 µM retinoic acid for a duration of 7 days to induce their differentiation into dopaminergic neuron‐like cells. They were incubated at 37°C in an incubator set to have 95% humidity and 5% CO_2_. Cells grew to approximately 70% confluency before they were used for experiments. We used commercially available escin, produced by Shandong Luye Pharmaceutical Co. Ltd., with the product batch number 230304405.

SH‐SY5Y cells were divided into the following groups: *Pg*‐LPS 10 µg/mL (*Pg*‐LPS10), *Pg*‐LPS + 2.5 µM escin (escin2.5), *Pg*‐LPS + 5 µM escin (escin5), and *Pg*‐LPS + 10 µM escin (escin10). Control group was grown in standard medium. The *Pg*‐LPS10 group was treated with *Pg*‐LPS (10 µg/ml final concentration). The escin dose groups were treated with various concentrations of escin for 120 min prior to *Pg*‐LPS treatment (10 µg/mL final concentration). Forty‐eight hours after *Pg*‐LPS exposure, the cells or the supernatant were harvested for assays.

To confirm escin's mode of action, the apoptosis inducer, staurosporine, was applied to the SH‐SY5Y cells. Cells were assigned into control, *Pg*‐LPS 10, escin, and escin + staurosporine groups. The escin + staurosporine group was pretreated with 10 µM escin for 2 h, followed by exposure to both *Pg*‐LPS (10 µg/mL final concentration) and 2 µM staurosporine (Dave et al. 2003).

### 2.2 Cell Viability

SH‐SY5Y cells (5000/well) underwent exposure with *Pg*‐LPS at doses ranging from 0, 1, 2, 5, or 10 µg/mL, for a duration of 24 h at 37°C (Ishida et al. 2017). Following incubation, 10 µL of MTT at 5 mg/mL concentration was introduced per well, then incubated for 4 h before 150 µL of dimethyl sulfoxide was added. Absorbance at 490 nm was assessed using a SpectraMax Paradigm Microplate Reader (M3 MD, USA). The effect of escin at 0, 5, 10, 20, 40, 80, and 160 µM on cells or *Pg*‐LPS‐challenged cells was also investigated.

### 2.3 Detection of Apoptosis

#### 2.3.1 JC‐1 Staining

Cells followed the procedures outlined in Section 2.1, were transferred to 12‐well plates with a density of 0.3 × 10^6^ cells/well, and were cultured for a duration of 12 h. The same volume of JC‐1 staining solution (prepared with distilled water at a 1:4 ratio and precooled on ice) and culture medium was then added to wells, mixed, and incubated in the dark at 37°C for 20 min. After discarding the staining solution, the cells underwent two rinses in JC‐1 medium prior to being analyzed with a confocal microscope (LSM800 Zeiss, Germany).

#### 2.3.2 Hoechst 33342/Propidium Iodide (PI) Staining

Cells followed the procedures outlined in Section 2.1, were transferred to 24‐well plates with a density of 2.0 × 10^4^ cells per well, and were cultured for a duration of 48 h. Following phosphate‐buffered saline (PBS) washing, cells underwent fixation for 15 min in 4% paraformaldehyde and were stained with Hoechst 33342/PI for an additional 5 min. The cellular specimens were meticulously observed and imaged utilizing an inverted fluorescence microscope (Olympus IX73, Japan).

#### 2.3.3 TUNEL Staining

Cells on glass slides were placed into wells of 24‐well plates (0.4 × 10^6^ cells/well) and subjected to the 48‐h culture process outlined in Section 2.1. The cell samples were meticulously washed twice with PBS to remove any residual debris or contaminants, ensuring a clean cellular environment for subsequent procedures. Following this, a precise volume of 100 µL of TUNEL reagent was introduced into each well to facilitate the detection of DNA fragmentation, a hallmark of apoptotic cells. Concurrently, 100 µL of PI dye solution was added to each well to enable the identification of necrotic or late‐stage apoptotic cells through its ability to intercalate into double‐stranded DNA. The cultures were meticulously maintained at a constant temperature of 37°C within a controlled environment shielded from light for a duration precisely measured as 1 h. After that, the cultures were then subjected to a series of rinsing steps. They were carefully rinsed three times with PBS. The glass slides were subsequently sealed with an anti‐fluorescence quenching agent and examined through a confocal microscope (LSM800 Zeiss, Germany).

#### 2.3.4 Flow Cytometry

SH‐SY5Y cells were cultivated in 12‐well plates (3 × 10^5^ cells/well) and exposed to *Pg*‐LPS for 24 h. The escin groups were treated with various concentrations of escin for 120 min prior to *Pg*‐LPS treatment (10 µg/mL final concentration). The medium was subsequently removed, and the cells underwent two washes with PBS. Cells were then digested with trypsin (without EDTA) and then treated with 5 µL Annexin V‐FITC or with 5 µL PI as a negative control. Annexin V‐FITC and PI single staining were performed for compensation adjustment. Cells were incubated in darkness at ambient temperature for 15 min before undergoing flow cytometric analysis (CytoFLEX S Beckman, USA).

### 2.4 RT‐PCR

SH‐SY5Y cells were seeded in 12‐well plates at a density of 0.3 × 10^6^ cells/well, cultured until 70% confluent, and then treated with *Pg*‐LPS and escin as described. The Ultrapure RNA kit (CWBIO, Beijing, China) was used for RNA extraction, which was then converted to cDNA. The following primers were used for PCR amplification: *BAX*, forward primer: 5′‐AAGCATTGAGAGGTG‐3′, reverse primer: 5′‐AGAGGAAGTGAGGAGAA‐3′; *BCL2*, forward primer: 5′‐GTTCCACCCGTTTTCA‐3′, reverse primer: 5′‐GCGAGTCCTCATTCTGT‐3′. PCR reactions consisted of 10 µL 2×QuantiNova SYBR Green RT‐PCR MasterMix, 20 ng cDNA, 0.6 µL 10 µmol/L forward primer, 0.6 µL 10 µmol/L reverse primer, and RNase‐free water. The reaction's total volume is 20 µL. The PCR protocol consisted of an initial step at 95°C for 2 min, then 40 cycles of 95°C for 5 s and 60°C for 31 s. A melting curve was produced using conditions of 95°C for 15 s, 60°C for 1 min, a gradual increase of 0.3°C/s to 95°C, 95°C for 30 s, and 60°C for 15 s. To determine the levels of *BAX* and *BCL2* mRNA, the 2^−ΔΔCt^ approach was applied.

### 2.5 Western Blotting

Cells were seeded in 6‐well plates at a density of 1 × 10^6^ cells per well, harvested via trypsinization, and lysed in RIPA buffer (50 mM Tris–HCl, pH 7.4, 150 mM NaCl, 1% NP‐40, 0.1% SDS, and 1 mM EDTA) supplemented with a protease inhibitor cocktail. Protein concentrations were quantified using the BCA assay. Equal amounts of protein (30 µg) were separated by 10% SDS–PAGE, transferred to PVDF membranes, and blocked with 5% BSA. The membranes were probed with primary antibodies (all from Affinity, Changzhou, China) against *BAX* (1:1000), *BCL*‐2 (1:1000), cleaved Caspase 3 (1:1000), Caspase 3 (1:1000), PARP (1:1000), and β‐actin (1:1000) at 4°C overnight, followed by incubation with HRP‐conjugated secondary antibody (1:1000, Beyotime) at room temperature for 1 h. β‐Actin served as a loading control on the same blot.

### 2.6 Effect of an Apoptotic Inducer on the Protective Action of Escin

To investigate whether escin could counteract apoptosis, SH‐SY5Y cells were pretreated with escin (10 µM) for 2 h, followed by simultaneous exposure to *Pg*‐LPS (10 µg/mL) and the apoptosis inducer staurosporine (2 µM), as described by Dave et al. (2003). This experimental group was designed to evaluate the protective effect of escin against staurosporine‐induced apoptosis under inflammatory conditions.

### 2.7 Statistical Analysis

Statistical analyses were conducted using GraphPad Prism software version 8.0 (GraphPad Software, MA, USA). The Shapiro–Wilk test was used to validate the normal distribution of data. Data are represented as the mean ± standard deviation. One‐way analysis of variance was utilized for subsequent comparisons, and Tukey's method was applied for follow‐up adjustments. Statistical significance was set at a level of *p* < 0.05.

## 3 Results

### 3.1 Effect of Escin on *Pg*‐LPS‐Induced Injury in SH‐SY5Y Cells

**FIGURE 1:**
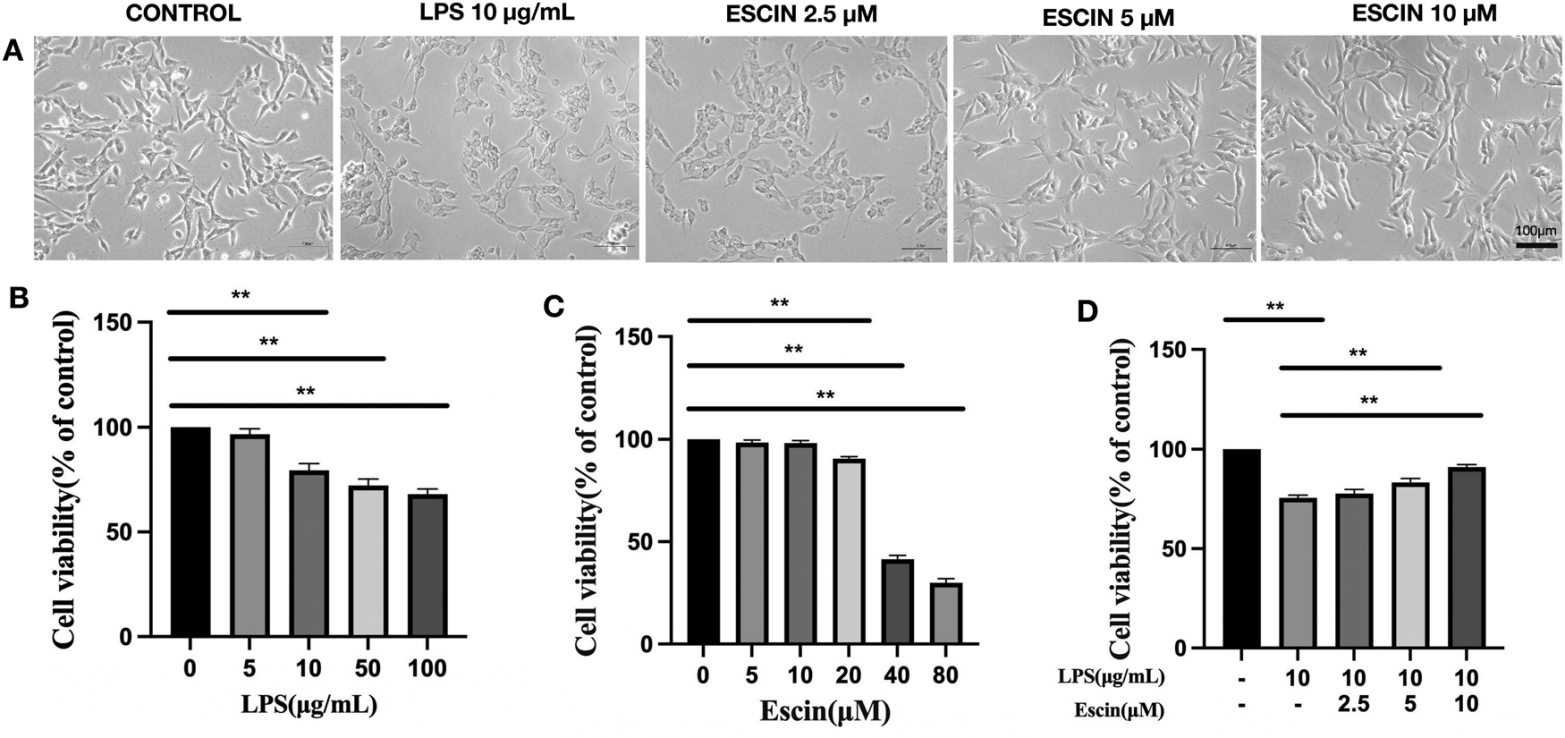
Effect of escin on *Pg*‐LPS‐induced injury to SH‐SY5Y cells. (A) Representative images of SH‐SY5Y cells treated with different concentrations of *Pg*‐LPS/escin. Bar = 100 µM; (B) viability of SH‐SY5Y cells treated with *Pg*‐LPS as indicated for 48 h; (C) viability of SH‐SY5Y cells treated with escin as indicated for 48 h; (D) viability of SH‐SY5Y cells treated with different concentrations of *Pg*‐LPS/escin as indicated for 48 h. Data are represented as the mean ± SD of three independent experiments. ^*^ *p *< 0.05, ^**^ *p *< 0.01.

A notable fraction of SH‐SY5Y cells in the *Pg*‐LPS group showed signs of injury. These included disorganized cell patterns, compromised structural integrity, shrunken cytoplasm, and enlarged cell bodies (Figure 1A). At concentrations exceeding 10 µg/mL, *Pg*‐LPS reduced SH‐SY5Y cell viability dose‐dependently (*p *< 0.05, Figure 1B). Escin at 2.5–10 µM showed no impact on SH‐SY5Y cell viability. However, escin levels exceeding 20 µM decreased SH‐SY5Y cell viability (*p* < 0.01, Figure 1C), which is consistent with literature research reports (Çiftçi et al. 2015; Zhu et al. 2017). Escin at 2.5–10 µM mitigated the reduction in SH‐SY5Y cell viability caused by *Pg*‐LPS (10 µg/mL) (*p* < 0.05; Figure 1D). Therefore, in subsequent experiments, *Pg*‐LPS was used at 10 µg/mL and escin at 2.5, 5, and 10 µM.

Although 10 µg/mL LPS significantly reduced SH‐SY5Y cell viability, this concentration was selected for subsequent experiments as it consistently induced a reproducible inflammatory and cytotoxic response while preserving sufficient cell survival (∼70%) for pharmacological intervention. This level of injury was deemed optimal to evaluate the potential protective effects of escin. Similar concentrations have been commonly used in SH‐SY5Y‐based inflammatory models (Ishida et al. 2017).

### 3.2 Effect of Escin on *Pg*‐LPS‐Induced Apoptosis of SH‐SY5Y Cells

**FIGURE 2:**
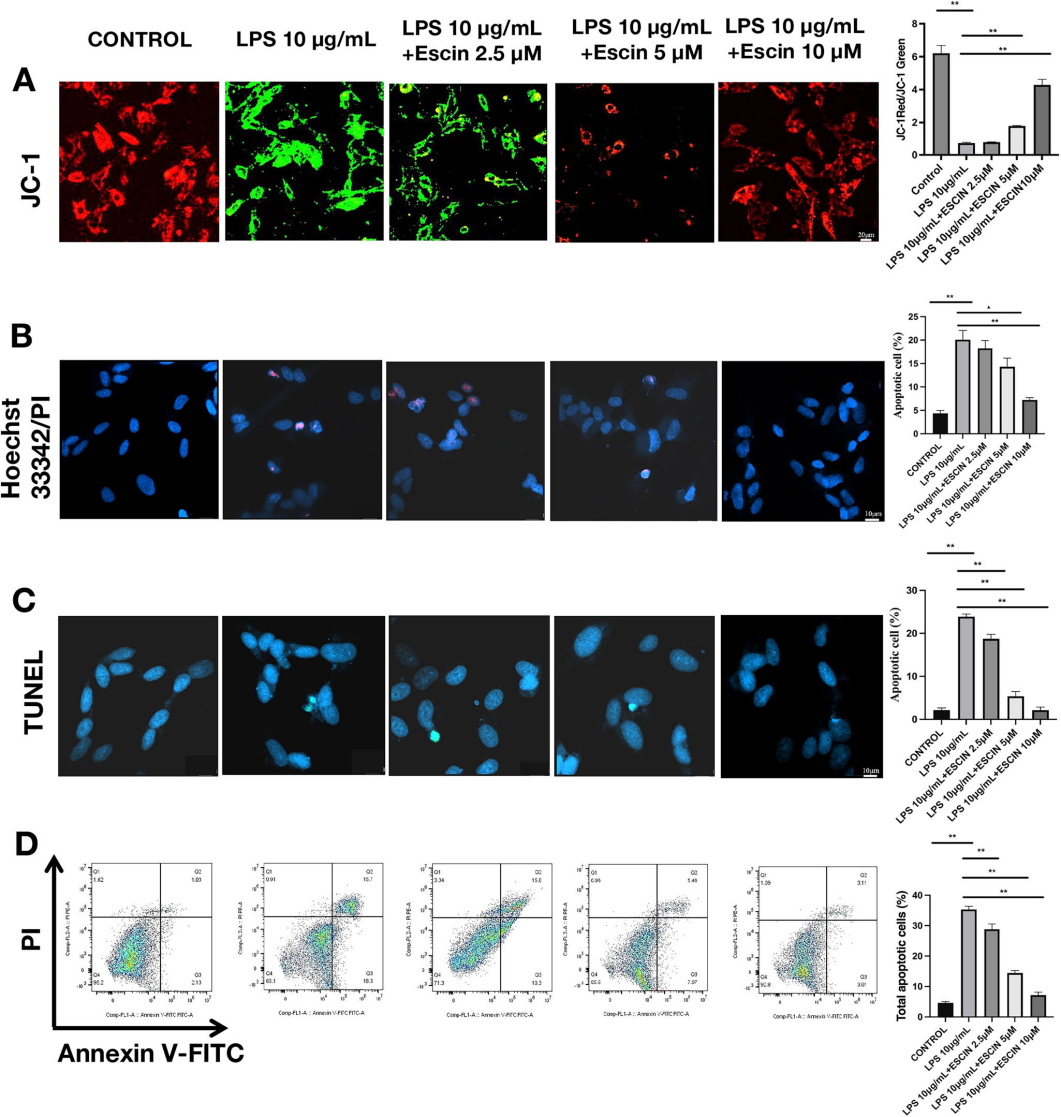
Effect of escin on *Pg*‐LPS‐induced apoptosis of SH‐SY5Y cells. (A) JC‐1 staining; (B) Hoechst 33342/PI staining; (C) TUNEL staining; (D) flow cytometry analysis. Apoptosis of SH‐SY5Y cells treated with different concentrations of *Pg*‐LPS/escin as indicated for 48 h. Bar = 20 µM. Data are represented as the mean ± SD of three independent experiments. ^*^ *p *< 0.05, ^**^ *p *< 0.01.

A decrease in mitochondrial membrane potential (MMP) is a key point in the initial stages of mitochondrial malfunction and apoptosis. Green fluorescence from JC‐1 staining indicates minimal MMP, representing MMP disruption, whereas red fluorescence from JC‐1 staining indicates high MMP. *Pg*‐LPS augmented the quantity of green fluorescent‐positive cells, whereas 2.5 µM escin notably enhanced the number of red fluorescent‐positive cells in a dose‐dependent manner (*p* < 0.05 for *Pg*‐LPS 10 µg/mL + escin 5 µM and *Pg*‐LPS 10 µg/mL + escin 10 µM groups compared to *Pg*‐LPS 10 µg/mL, Figure 2A). Hoechst 33342 emits blue fluorescence upon interacting with DNA, whereas PI selectively labels dead cells and emits red fluorescence because of its ability to penetrate damaged cell membranes. *Pg*‐LPS increased the number of condensed nuclei and PI‐positive cells in Hoechst 33342/PI staining, an effect that was dose‐dependently mitigated by escin (*p* < 0.05, Figure 2B). TUNEL staining and flow cytometry also showed that *Pg*‐LPS caused significant apoptosis of SH‐SY5Y cells and that escin reduced *Pg*‐LPS‐triggered apoptosis of SH‐SY5Y cells in a dose‐dependent manner (*p* < 0.05, Figure 2C,D).

### 3.3 Effect of Escin on *Pg*‐LPS‐Induced Apoptosis‐Related Signaling

**FIGURE 3:**
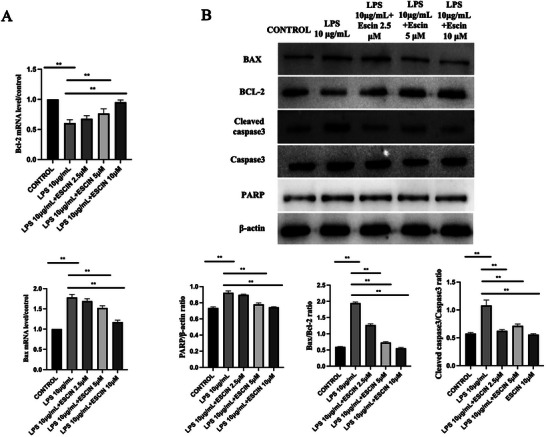
Effect of escin on apoptosis signaling in *Pg*‐LPS‐treated SH‐SY5Y cells. (A) *BAX* and *BCL2* mRNA levels; (B) protein levels of *BAX*, *BCL2*, cleaved Caspase 3, Caspase 3, and PARP. Apoptosis of SH‐SY5Y cells treated with different concentrations of *Pg*‐LPS/escin as indicated for 48 h. Data are represented as the mean ± SD of three independent experiments. ^**^ *p *< 0.01.

MRNA levels of *BAX* were elevated, and those of *BCL2* were decreased in *Pg*‐LPS‐challenged SH‐SY5Y cells (*p* < 0.05). Escin diminished *BAX* while concurrently enhancing *BCL2* mRNA levels (*p* < 0.05, Figure 3A). Exposure to *Pg*‐LPS markedly down‐regulated *BCL2* protein levels in SH‐SY5Y cells. In contrast, the levels of cleaved Caspase 3, *BAX*, Caspase 3, and PARP were upregulated after exposure to *Pg*‐LPS (Figure 3B). Escin attenuated the apoptotic effect induced by *Pg*‐LPS (Figure 3B). Escin increased *BCL2* levels while reducing those of cleaved Caspase 3, *BAX*, Caspase 3, and PARP. The *BAX/BCL2* ratios demonstrated that escin protected SH‐SY5Y cells from *Pg*‐LPS‐induced apoptosis (*p* < 0.05). Escin decreased the cleaved Caspase 3/Caspase 3 ratio (*p* < 0.05 for all escin‐treated groups compared to *Pg‐*LPS 10 µg/mL) and the level of PARP significantly (*p* < 0.05 for *Pg*‐LPS 10 µg/mL + Escin 5 µM and *Pg*‐LPS 10 µg/mL + Escin 10 µM groups compared to *Pg*‐LPS 10 µg/mL).

### 3.4 Effect of Staurosporine on the Anti‐Apoptotic Activity of Escin in SH‐SY5Y Cells

**FIGURE 4:**
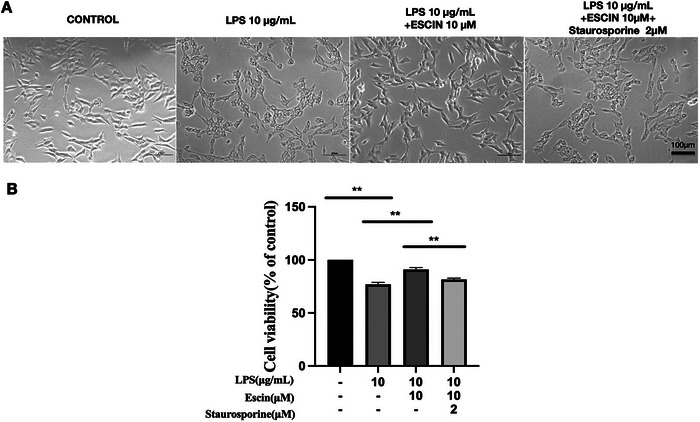
Effect of staurosporine on escin‐attenuation of *Pg*‐LPS‐induced SH‐SY5Y cell injury. (A) Representative images of SH‐SY5Y cells treated with different concentrations of *Pg*‐LPS, escin, and staurosporine. Bar = 100 µM. (B) Viability of SH‐SY5Y cells treated with *Pg*‐LPS, escin, and staurosporine as indicated for 48 h. Data are represented as the mean ± SD of three independent experiments. ^##^ *p *< 0.01 compared with the control group. ^*^ *p *< 0.05, ^**^ *p *< 0.01.

Staurosporine is a well‐established apoptotic inducer. When SH‐SY5Y cells were co‐treated with escin and staurosporine following *Pg*‐LPS exposure, significant morphological alterations indicative of apoptosis were observed, including cytoplasmic shrinkage, loss of structural integrity, and enlarged, rounded cell bodies (Figure 4A). Co‐treatment significantly reduced the escin‐mediated increase in cell viability (*p* < 0.05, Figure 4B), suggesting that staurosporine partially counteracts the cytoprotective effect of escin.

**FIGURE 5:**
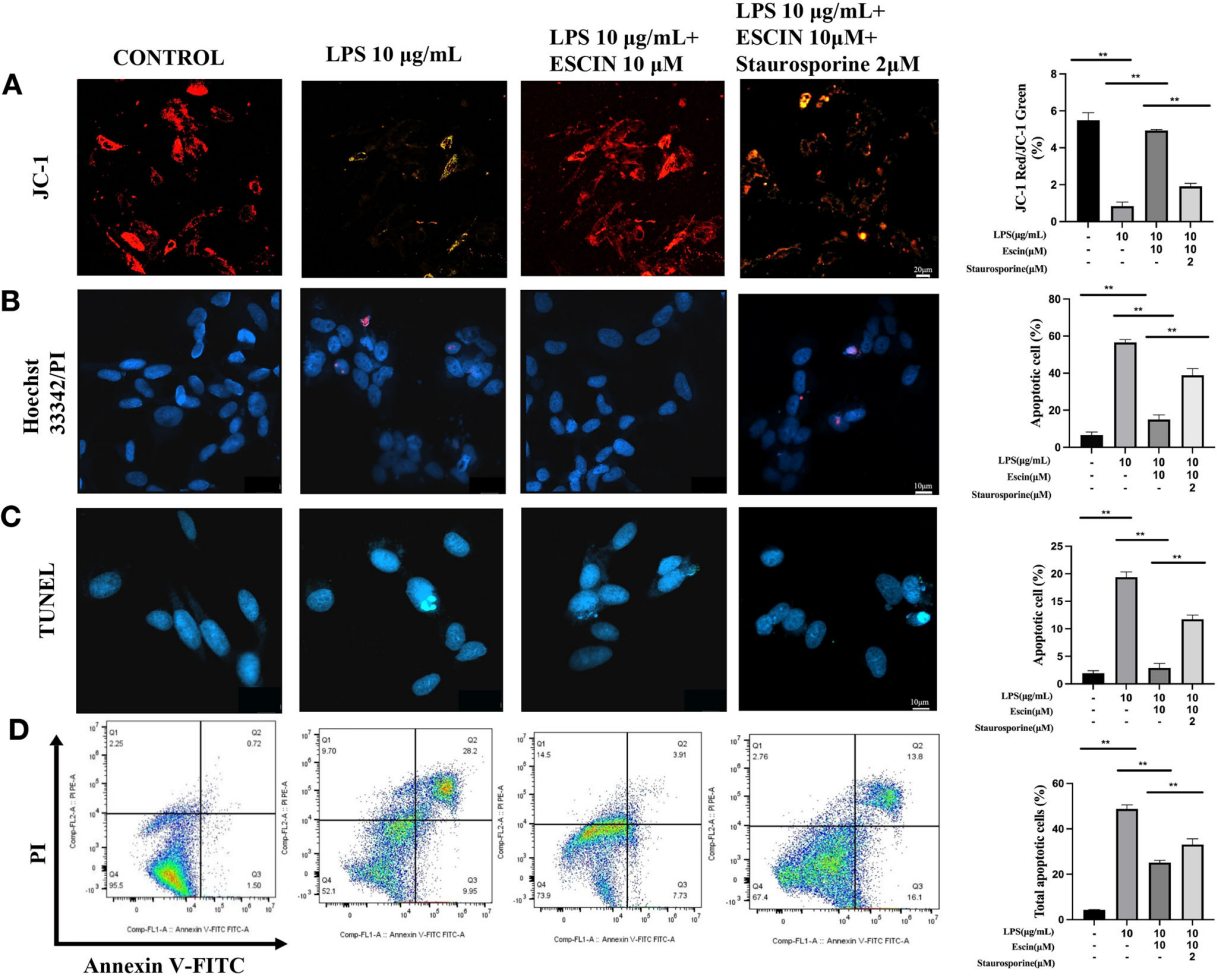
Effects of staurosporine on escin‐attenuation of *Pg*‐LPS‐induced SH‐SY5Y cell apoptosis. (A) JC‐1 staining; (B) Hoechst 33342/PI staining; (C) TUNEL staining; (D) flow cytometry analysis. Apoptosis of SH‐SY5Y cells treated with different concentrations of *Pg*‐LPS, escin, and staurosporine as indicated for 48 h. Bar = 20 µM. Data are represented as the mean ± SD of three independent experiments. ^*^ *p *< 0.05, ^**^ *p *< 0.01.

**FIGURE 6:**
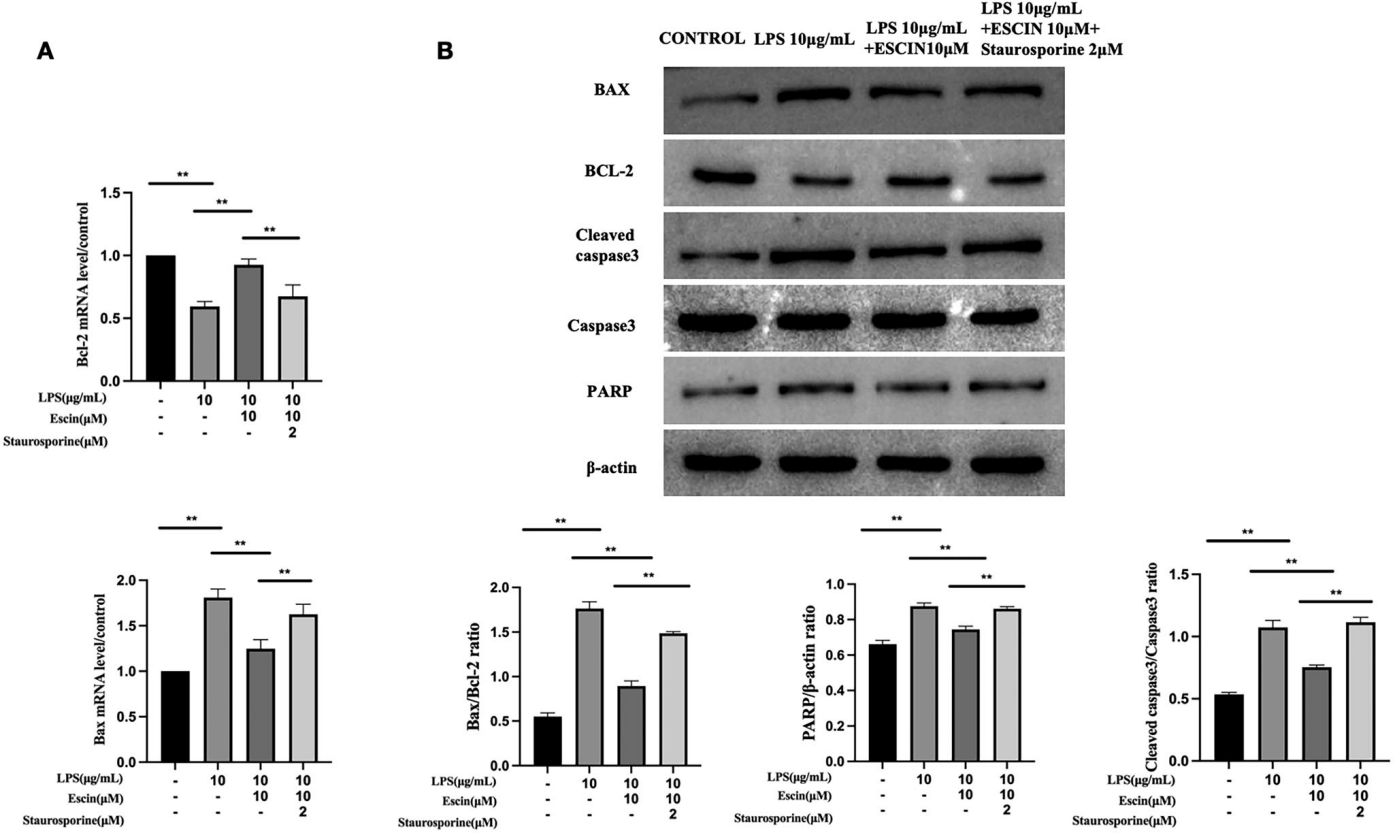
Effects of staurosporine on escin‐attenuation of *Pg*‐LPS‐induced SH‐SY5Y cell apoptotic signaling. (A) *BAX* and *BCL2* mRNA levels. (B) Protein levels of *BAX*, *BCL2*, cleaved Caspase 3, Caspase 3, and PARP. Apoptosis of SH‐SY5Y cells treated with different concentrations of *Pg*‐LPS/escin as indicated for 48 h. Data are represented as the mean ± SD of three independent experiments. ^**^ *p *< 0.01.

Furthermore, flow cytometric and fluorescence‐based assays (JC‐1, Hoechst 33342/PI, and TUNEL) revealed that staurosporine markedly increased the percentage of apoptotic cells despite escin pre‐treatment, effectively negating its anti‐apoptotic effect (*p* < 0.05, Figure 5). At the molecular level, staurosporine significantly upregulated *BAX* and down‐regulated *BCL2* mRNA expression (*p* < 0.05, Figure 6A), indicating an attenuation of escin's modulatory influence on apoptotic gene expression Supporting Material.

Consistent with these findings, Western blot analysis demonstrated that staurosporine reversed the escin‐induced modulation of key apoptosis‐related proteins, including *BCL2*, cleaved Caspase 3, *BAX*, and cleaved PARP, in *Pg*‐LPS‐treated SH‐SY5Y cells (*p* < 0.05, Figure 6B). These results collectively support the notion that escin exerts its protective effects, at least in part, through anti‐apoptotic mechanisms, which are compromised in the presence of a potent apoptosis inducer such as staurosporine.

## 4 Discussion

*P. gingivalis* is a common bacterium that causes chronic periodontitis. LPS is one of the ingredients of *P. gingivalis*, and it is inevitably the cardinal pathogenic factor associated with PD. *Pg*‐LPS increased the activation of astrocytes and microglia in the hippocampus, significantly increased the expression of IL‐1 β and TNF‐α, and reduced the activity of hippocampal neurons in mice, leading to depressive‐like behavior (Li et al. 2023). Neuroinflammation is a prominent pathological hallmark of PD. Multiple independent studies have validated LPS‐induced SH‐SY5Y cells as a relevant model for investigating PD‐associated neurotoxicity and neuroprotective mechanisms (Ling et al. 2021; Niaz et al. 2021; Wu et al. 2020; Zang et al. 2022; Zheng et al. 2024). Therefore, the LPS‐induced SH‐SY5Y cell model was employed to investigate the effect of *P. gingivalis* on PD and the impact of escin on *Pg*‐LPS‐triggered apoptosis. *Pg*‐LPS caused cell injury and induced apoptosis in SH‐SY5Y cells, whereas escin reversed this injury and protected against apoptosis. *Pg*‐LPS activated apoptosis factors, such as cleaved Caspase 3, *BAX*, and PARP, while concurrently reducing *BCL2* levels, which is consistent with previous reports highlighting the toxic effects of bacterial LPS on neuronal cells (Nativel et al. 2017). Conversely, escin diminished the upregulation of these apoptotic factors. In a rat's model with superior sagittal sinus thrombosis (SSST), escin treatment for 7 consecutive days improved BBB disruption and protected tight junction proteins, the number of microglia‐originated macrophages was decreased, and the NLRP3, Caspase 1, IL‐1β, IL‐18, and GSDMD protein expression levels were down‐regulated in the parasagittal cortex, indicating its anti‐pyroptosis and neuroprotective effect (Li et al. 2025). Staurosporine, an apoptosis inducer, further confirmed the anti‐apoptotic effect of escin. Therefore, *Pg*‐LPS contributes to mitochondrial impairment and apoptosis in SH‐SY5Y cells, and escin exerts an inhibitory effect on *Pg*‐LPS‐induced apoptosis.

*P. gingivalis* infection can increase reactive oxygen species production and impair mitochondrial function, as has been revealed in the HAEC cell model (Xu et al. 2025). Treatment cells with *P. gingivalis* may generate numerous responses, such as neuroinflammation, apoptosis, oxidative stress, and disruption of cellular homeostasis, which can mirror the oxidative stress and mitochondrial dysfunction observed in PD and may provide insights into the mechanisms by which this periodontal pathogen contributes to PD (Verma et al. 2023; Yamada et al. 2020). This would help bridge the gap between periodontal disease and neurodegenerative disorders (Chen et al. 2017).

MMP is recognized as a pivotal factor in the etiology of PD. JC‐1 staining demonstrated MMP impairment and apoptosis in *Pg*‐LPS‐treated SH‐SY5Y cells, indicating a causal relationship between *Pg*‐LPS and MMP impairment. However, the MMP impairment and apoptosis were inhibited by escin treatment. Hoechst 33342/PI and TUNEL staining with flow cytometry confirmed the ability of escin to counteract or reduce *Pg*‐LPS‐induced apoptosis in SH‐SY5Y cells. These findings showed that *Pg*‐LPS may contribute to dopaminergic neuron damage in PD and that escin mitigated the injury and apoptosis caused by *Pg*‐LPS in SH‐SY5Y cells.

This study reveals how *Pg*‐LPS triggers mitochondria‐mediated apoptosis in SH‐SY5Y cells. Specifically, the administration of *Pg*‐LPS dramatically decreased the *BCL2/BAX* ratio. This is likely to cause increased mitochondrial membrane permeability, which in turn facilitates the discharge of cytochrome C from mitochondria into the cytoplasm (Li et al. 2020). This phenomenon aligns with the regulatory role of the *BCL2* protein family in the classical apoptotic pathway, where the anti‐apoptotic protein, *BCL2*, maintains mitochondrial membrane stability by inhibiting *BAX* oligomerization, whereas *BAX* activation triggers mitochondrial membrane permeability and initiation of the apoptotic cascade (Czabotar et al. 2014). The *BCL2* family, comprising *BCL2* and *BAX*, regulates the mitochondria‐mediated apoptosis process. *BAX* is triggered during the pro‐apoptotic phase, whereas *BCL2* exerts an anti‐apoptotic influence (Lin et al. 2018). The balance of *BAX* to *BCL2* influences cell stability and viability. Should the equilibrium be disrupted by exogenous factors, programed cell death will ensue (Elmore 2007). The reduction in the *BCL2/BAX* ratio in this study further supports the hypothesis that *Pg*‐LPS drives apoptosis through the mitochondrial pathway. Moreover, *Pg*‐LPS significantly increased the levels of active Caspase 3 and cleaved PARP, highlighting the pivotal role of Caspase 3 activation in apoptosis. Caspase 3 is an “executioner caspase” that disrupts DNA repair mechanisms by cleaving substrates such as PARP, ultimately leading to apoptosis (Elmore 2007).

This modulation of apoptotic genes underscores the importance of gene regulatory mechanisms in neuronal cell fate determination and highlights a potential role of escin as a neuroprotective agent through gene expression modulation. These discoveries shed light on the complex relationship between cell death mechanisms and neuronal survival and indicate the potential for precision treatments that can boost protective cellular responses (Suttkus et al. 2016). Therefore, it is reasonable to conclude that escin exerted its anti‐apoptotic effects by regulating the *BCL2*/*BAX*/Caspase 3 pathway.

To better understand how the effects of escin are mediated, we examined whether introducing a known apoptosis trigger would counteract the protective effects of escin against *Pg*‐LPS‐induced damage and cell death in SH‐SY5Y cells. This experiment aimed to determine if the therapeutic benefits of escin could be undermined by programed cell death pathways. Staurosporine, a specific inducer of apoptosis, activates caspases and other apoptotic pathways (Sarkar et al. 2024). Combining staurosporine with escin markedly decreased cell viability while simultaneously boosting rates of apoptosis. Additionally, the treatment upregulated key apoptotic markers, including the *BCL2*/*BAX* ratio, Caspase 3 activity, and PARP cleavage. These findings further confirmed that escin protected SH‐SY5Y cells through anti‐apoptotic effects. In MPTP/*p*‐induced mouse model of PD, previous studies demonstrated that escin ameliorated the injury of dopaminergic neurons abnormalities of neurological function through its antioxidant and anti‐inflammatory properties. Furthermore, escin also attenuated MPTP/*p*‐induced mitochondrial dysfunction, oxidative stress, and apoptosis (Selvakumar et al. 2020). Relative to neurotoxins, *P. gingivalis* is a common bacterium in mouth cavity. Our findings reveal novel perspectives on the influence of *P. gingivalis* on PD. The results of this study demonstrate for the first time that escin has protective effects against *Pg*‐LPS. Therefore, the ability of escin to counteract *Pg*‐LPS‐induced apoptosis highlights its potential as a protective agent for preventing PD.

Specific limitations of our study requiring consideration in subsequent studies should be highlighted. First, SH‐SY5Y cell line is a simple, easy in vitro model for studying PD. SH‐SY5Y cell imitates the injured dopaminergic neurons. In addition, it is similar to dopaminergic neurons, which are sensitive to LPS or neurotoxins. However, SH‐SY5Y cell line is not considered an authentic dopaminergic cell line. The cell line has some disadvantages such as the possibility of turning into an epithelial phenotype and the presence of genetic instability. Nevertheless, SH‐SY5Y cell is useful as a first step for testing potential interventions in experimental PD. Therefore, the present findings of this study should be further expanded to more complex in vitro models such as primary neurons or to in vivo murine PD models. Second, other potential mechanisms such as oxidative stress, NF‐κB‐mediated inflammation, or mitochondrial biogenesis may contribute to the *Pg*‐LPS‐induced injury of dopaminergic neurons, and therefore more detailed studies about the mechanism of action of escin are needed.

## 5 Conclusion

In conclusion, we demonstrated the induction of mitochondrial malfunction and apoptosis in *Pg*‐LPS‐treated SH‐SY5Y cells. Escin reduced *Pg*‐LPS‐induced SH‐SY5Y cell injury by inhibiting apoptosis. Escin exerted its anti‐apoptotic effects by affecting the *BCL2*/*BAX*/Caspase 3 pathway.

## Author Contributions

**Yingying Sun**: writing – original draft, writing – review and editing, methodology, formal analysis. **Rui Zhang**: validation, investigation, data curation. **Yunping Pan**: software, investigation, data curation. **Yufei Sun**: methodology, data curation, software. **Limin Liu**: methodology, data curation, resources. **Min Feng**: software, visualization, project administration. **Yuan Du**: methodology, investigation, writing – review and editing. **Tian Wang**: writing – review and editing. **Shutai Liu**: conceptualization, funding acquisition, supervision, writing – review and editing.

## Conflicts of Interest

The authors declare no conflicts of interest.

## Permission to Reproduce Material From Other Sources

The figures in the manuscript were not from other sources.

## Peer Review

The peer review history for this article is available at https://publons.com/publon/10.1002/brb3.70810.

## Supporting information




**Supporting Material**: brb370810‐sup‐0001‐SuppMatt.pdf

**Supporting Material**: brb370810‐sup‐0002‐SuppMatt.pdf

## Data Availability

The data that support the findings of this study can be obtained from the corresponding author upon reasonable request.
