# Supplementary material for: Anti‐Apoptotic Effects of Escin on Porphyromonas gingivalis–Derived Lipopolysaccharide‐Induced Injury in SH‐SY5Y Cells
Source: Brain Behav. 2025 Sep 2;15(9):e70810. doi: 10.1002/brb3.70810 (PMC12402593; doi:10.1002/brb3.70810)
Supplement: Supplementary file 1 — Supporting Material: brb370810‐sup‐0001‐SuppMatt.pdf [file BRB3-15-e70810-s002.pdf]

SH-SY5Y [SHSY-5Y]细胞 STR 鉴定报告

一、 材料处理和检验方法

取适量 **SH-SY5Y [SHSY-5Y]** 细胞(编号 PNS-HC-80,1×10^6)使用 Chelex100 提取 DNA，采用 21 CELLID System 扩增 20 个 STR 位点和性别鉴定位点，使用 ABI3130x1 型遗传分析仪进行 PCR 产物检测，使用 GeneMapper IDX 软件（Applied Biosystems）对检测结果进行分析，并与 ATCC、DSMZ、JCRB、Cellosaurus 等数据库进行比对。

二、 检测结果

实验中阴性及阳性对照结果均正确。

SH-SY5Y [SHSY-5Y] 细胞株的 STR 位点和 Amelogenin 位点的基因分型结果见附表，分型图谱见附图。

三、 分析说明

SH-SY5Y [SHSY-5Y] 细胞株基因组 DNA 扩增后图谱清晰，分型结果良好。

四、 检验结论

1. **SH-SY5Y [SHSY-5Y]** 细胞株 DNA 进行细胞 STR 分型结果显示，细胞株中未发现人类细胞交叉污染。
2. 该细胞株 DNA 分型在细胞库中找到与其细胞分型 100.00%相匹配的细胞株，细胞株名称为 **SH-SY5Y [SHSY-5Y]**。

www.procell.com.cn Procell

附表 1：细胞株 **SH-SY5Y [SHSY-5Y]**的 STR 位点和 Amelogenin 位点的基因分型结果

STR Loci	样品名称：PNS-HC-80	数据库名称：SH-SY5Y [SHSY-5Y]
Amelogenin	X	X
CSF1PO	11	11
D2S1338	17,19	17,19
D3S1358	15,16	15,16
D5S818	12	12
D7S820	7,10	7,10
D8S1179	15	15
D13S317	11	11
D16S539	8,13	8,13
D18S51	13,16	13,16
D19S433	13,14	13,14
D21S11	31,31.2	31,31.2
FGA	23.2,24	23.2,24
PentaD	10,12	10,12
PentaE	7,11	7,11
TH01	7,10	7,10
TPOX	8,11	8,11
vWA	14,18	14,18
D6S1043	12,18	12,18
D12S391	18,22	18,22
D2S441	11,11.3	11,11.3
Cellosaurus 数据库匹配度 100.00%，匹配位点数 20（https://web.expasy.org/cellosaurus-str-search/）		

Procell www.procell.com.cn

备注：

1. **根据国际细胞鉴定委员会(ICLAC)制定的细胞 STR 鉴定标准，细胞系的匹配度≥80% 时，认为它们具有相关性，即衍生于共同的祖先细胞；匹配度在 55% 至 80% 之间，需要进一步验证相关性；小于 55%，表明两者不具有相关性。**
2. **图谱有效峰为真实的 PCR 条带;小峰和非特异性条带在计算中忽略不计。**
3. **STR 数据比对结果默认 ExPASy，数据来源包括 ATCC，DSMZ，JCRB 等细胞库以及文献和资料记载，数据库入口 https://web.expasy.org/cellosaurus-str-search/。**

Copyright ©2019-2020 Procell Life Science&Technology Co.,Ltd. All Rights Reserved

武汉普诺赛生命科技有限公司

Procell Life Science&Technology Co.,Ltd.

附图 1：SH-SY5Y [SHSY-5Y]细胞 STR 位点和 Amelogenin 位点的基因分型结果

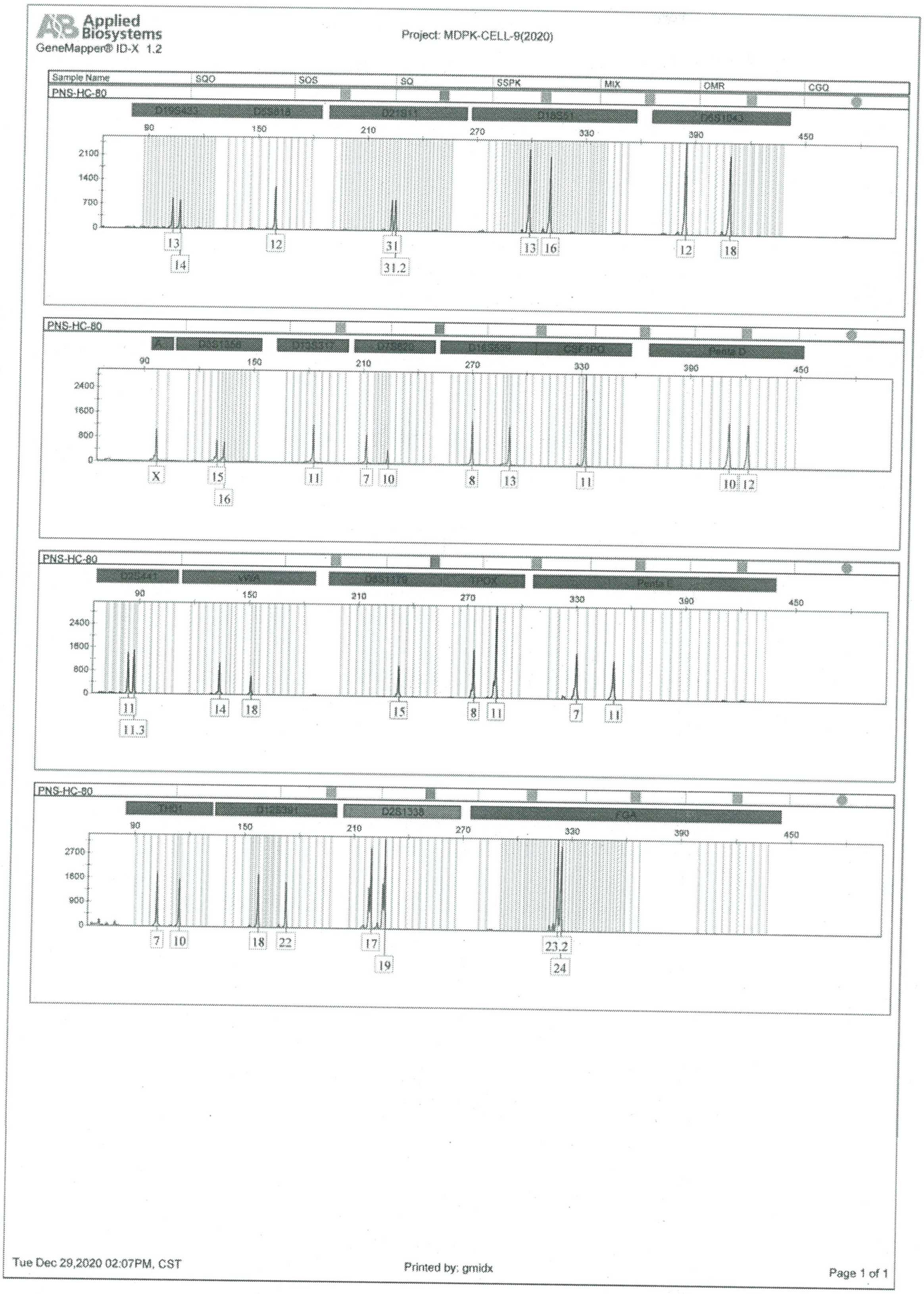

销售电话：400-650-3656
企业 QQ：4006503656
销售邮箱：sales@procell.com.cn
官方网站：www.procell.com.cn
