# Supplementary material for: Anti‐Apoptotic Effects of Escin on Porphyromonas gingivalis–Derived Lipopolysaccharide‐Induced Injury in SH‐SY5Y Cells
Source: Brain Behav. 2025 Sep 2;15(9):e70810. doi: 10.1002/brb3.70810 (PMC12402593; doi:10.1002/brb3.70810)
Supplement: Supplementary file 2 — Supporting Material: brb370810‐sup‐0002‐SuppMatt.pdf [file BRB3-15-e70810-s001.pdf]

普诺赛® Procell | Pricella

武汉普诺赛生命科技有限公司
Wuhan Pricella Biotechnology Co., Ltd.

# Certificate of Analysis

SH-SY5Y [SHSY-5Y]

人神经母细胞瘤细胞

货号：CL-0208 报告批准日期：2024-07-24

规格：1×10^6Cells/T25 保存温度：常温

## Quality Profile

| 检测项目 | 单位 | 标准 | 结果 |
|---|---|---|---|
| 外观 | / | 标签清晰正确，瓶身外观完好，瓶盖盖紧 | 合格 |
| 数量 | cell | ≥1×10^6 | 合格 |
| 汇合度 | / | ≥70% | / |
| 细胞形态 | / | 上皮细胞样 | 合格 |
| 细菌 | / | 镜检无污染 | 合格 |
| 真菌 | / | 镜检无污染 | 合格 |
| 支原体 | / | PCR法/显色法阴性 | 合格 |

**质检结论：**

参考《SH-SY5Y [SHSY-5Y]细胞产品技术要求》，判定该产品**合格！**

武汉普诺赛生命科技有限公司 质检专用章

报告人：李丢

批准人：熊薇

网站: www.procell.com.cn
电话: 400-999-2100
邮箱: techsupport@procell.com.cn
地址: 湖北省武汉市高新大道858号生物医药产业园三期C4栋

2024-07-26

扫描全能王 创建
